# Urinary Tract Infections in Hospitalized Ischemic Stroke Patients: Source and Impact on Outcome

**DOI:** 10.7759/cureus.1014

**Published:** 2017-02-06

**Authors:** Einar Bogason, Kathy Morrison, Omar Zalatimo, David M Ermak, Erik Lehman, Eric Markley, Kevin Cockroft

**Affiliations:** 1 Department of Neurosurgery, Mercy Medical Center; 2 Penn State Hershey Stroke Center; 3 Department of Neurosurgery, Penn State Hershey Medical Center; 4 Department of Neurology, Penn State Hershey Medical Center; 5 Public Health Sciences, Penn State Hershey Medical Center

**Keywords:** urinary tract infections, ischemic stroke, outcomes, quality improvement

## Abstract

Background: Urinary tract infections (UTIs) in ischemic stroke patients are a common occurrence and the frequent focus of quality improvement initiatives. However, many UTIs are community-acquired and the impact of such infections on patient outcomes remains controversial.

Methods: We conducted a retrospective analysis of our Stroke Center Database and electronic medical records to determine the incidence of both community-acquired UTI (CA-UTI) and hospital-acquired UTI (HA-UTI) in hospitalized ischemic stroke patients. We assessed risk factors for UTI, as well as clinical outcome, the length of stay (LOS), and hospital charges.

Results: In our study sample of 395 patients, UTIs were found in 11.7% and the majority of these (65%) were found on admission. Patients admitted from another hospital were more likely to be diagnosed with a UTI of any type compared to those arriving from home (odds ratio (OR) 2.42 95%, confidence interval (CI) 1.18, 4.95) and were considerably more likely to have an HA-UTI than a CA-UTI (OR 12.06 95% CI 2.14, 95.32). Those with a Foley catheter were also more likely to have a UTI (OR 2.65 95% CI 1.41, 4.98). In the multivariable analysis, we did not find a statistically significant relationship between any UTI or a specific UTI subtype and discharge modified Rankin Score (mRS), LOS, or hospital charges. Admission stroke severity remained associated with higher odds of discharge in poor condition (adjusted odds ratio (aOR) 6.23 95% CI2.33, 16.62), an extended LOS (6.84 vs 5.07, p = 0.006), and higher hospital charges ($18,305 vs $12,162, p = 0.001).

Conclusions: Urinary tract infections remain a common occurrence in stroke patients. However, the majority of UTIs are present on admission and may have little impact on discharge clinical condition, LOS, or hospital charges. These results may have implications for quality improvement (QI) initiatives that focus on the prevention and treatment of hospital-acquired UTIs.

## Introduction

Stroke is one of the leading causes of morbidity and mortality in the Western world. The care of stroke patients often requires substantial resources not only during the acute phase but also after discharge since a considerable number of patients will require constant nursing care [1]. The complication rate in stroke patients is fairly high and is linked to stroke severity, with the most common complications being falls, skin breakdown, and infections. Pneumonia and urinary tract infections (UTIs) are the most frequent types of infections seen. The incidence of infections varies, but previous studies have quoted the rate for UTI as ranging from 3% to up to 40% [2-7]. Studies have suggested a link between infection and poor clinical outcome after stroke [8]. Elimination of certain infections, including UTIs, has also been tied to changes in reimbursement [9]. For this reason, many quality improvement (QI) programs have focused on reducing the number of infections and, as part of that process, determining which infections are hospital-acquired as opposed to community-acquired. The distinction implies that hospital providers can influence the incidence of hospital-acquired infections, but not of community-acquired ones. UTI, in particular, has been a popular topic of many QI initiatives, given its relatively high frequency; yet, it also presents a challenge since many patients meet UTI criteria on admission and the impact of an asymptomatic or minimally symptomatic UTI on a patient’s outcome is unclear. The goals of this study were to 1) access the incidence of both community and hospital-acquired UTI in our institution's stroke population and 2) to determine what effect, if any, the presence of UTI had on various outcome measures.

## Materials and methods

We conducted a retrospective analysis of our Stroke Center Database, along with our electronic medical records, over a two year period. Penn State Hershey Medical Center/Penn State College of Medicine Human Subjects Protection Office (HSPO) issued approval 35304EP for this study. Patient consent was obtained or waived at the time of treatment. All patients admitted with a diagnosis of acute ischemic stroke were initially reviewed. We gathered data concerning demographic information, location of patient prior to admission (e.g., home, hospital, nursing home), Trial of Org 10172 in Acute Stroke Treatment (TOAST) criteria [10], UTI occurrence, urinalysis and urine culture results, Foley placement, presence of sepsis [11], admission and discharge modified Rankin Scores (mRS), and admission and discharge National Institutes of Health Stroke Scale ( NIHSS). Admission mRS was determined based on historical information acquired from the patient, family, and medical record and reflects the condition of the patient prior to the stroke. A urinalysis (UA) was defined as positive if either nitrite or leukocyte esterase were positive. However, an actual UTI was defined as a positive urine culture. A community-acquired UTI (CA-UTI) was defined as being present on admission, while a hospital-acquired UTI (HA-UTI) was defined as being detected any time after admission. Admission and discharge NIHSS were dichotomized, with a score of > 10 being considered a severe stroke. Admission and discharge mRS were also dichotomized, with a score of three or more being considered poor condition/outcome and a score of two or less being a good baseline condition/functional outcome. The length of stay (LOS) and hospital charges were obtained from hospital records.

### Statistical methods

All variables were summarized initially with frequencies and percentages or means, medians, and standard deviations. Admission and discharge mRS and NIHSS were dichotomized at the upper quartile for the sake of analysis. Logistic regression was used to determine factors that are significantly associated with any UTI versus no UTI and with hospital-acquired UTI versus community-acquired UTI. Logistic regression was also used to examine the association between poor clinical outcome and other factors. Because their distributions were skewed and not normal in nature, length of stay and hospital charges were log-transformed prior to analysis. An Analysis of Variance (ANOVA) was then used to make comparisons between the means of the groups of other variables. The means and confidence limits resulting from the ANOVA were exponentiated back to their original units. Tukey’s method was used to adjust for multiple comparisons for variables with more than two groups. A multivariable model was applied to each outcome that included all variables from the bivariate analysis. The odds ratios and mean estimates from the multivariable models were adjusted for all other variables in the model. Multicollinearity was tested for between variables using variance inflation factors (VIF) statistics from linear regression, but none were found. The fit of the logistic regression model was checked using Deviance, Pearson, and Hosmer and Lemeshow goodness-of-fit statistics. All analyses were performed using SAS® 9.4 (SAS Institute, Cary, NC).

## Results

The initial review yielded 404 observations, but after limiting this to unique subjects based on the last admission date, we were left with 395 patients. The demographic information for the included patients can be seen in Table 1. 

**Table 1:** Overall Patient Characteristics mRS: modified Rankin Scale; NIHSS: National Institutes of Health Stroke Scale; SD: standard deviation; TOAST: Trial of Org 10172 in Acute Stroke Treatment; UA - urinalysis; WBC: white blood count

Variable (N = 395)	N (%) or Mean ± SD
Age (years)	68.5 ± 16.6
Female	182 (46.1)
Urinary tract infection	30 (7.6)
Community-acquired	16 (4.1)
Hospital-acquired	349 (88.4)
None	
Admission source	289 (73.2)
Home	81 (20.5)
Outside hospital	25 (6.3)
Nursing home	
Foley	122 (33.0)
UA positive results	52 (18.9)
UA positive nitrites	36 (12.8)
UA positive leukocytes	75 (26.5)
UA positive bacteria	185 (76.8)
UA positive sepsis	19 (4.8)
UA WBC in urine > 4	71 (18.0)
TOAST criteria	113 (28.6)
Large vessel	102 (25.8)
Small vessel	88 (22.3)
CardioEmbolic	65 (16.5)
Other determined	27 (6.8)
Undetermined	
Admission severe stroke (NIHSS ≥ 10)	79 (21.0)
Admission poor condition (mRS ≥ 3)	84 (22.3)
Discharge severe stroke (NIHSS ≥ 10)	61 (16.1)
Discharge poor condition (mRS ≥ 3)	127 (32.2)
Length of stay (days)	5.47 ± 4.10
Hospital charges ($)	13,660 ± 11,747

Urinary catheters were placed in 122 patients (33%) with the majority placed at our institution. The total UTI occurrence was 11.7%, with the majority of UTIs (65%) being present on admission. The mRS on admission was considered good (≤ 2) for 311 patients (78%) while, on discharge, the mRS was good for 268 patients (68%).

Results of a bivariate logistic regression for factors associated with UTI, or with CA-UTI versus HA-UTI in our study population, can be seen in Table 2. 

**Table 2:** Factors Associated with UTI Overall or with Hospital-acquired UTI Versus UTI on Admission * Odds ratios and p-values from logistic regression, exact logistic regression used if needed. mRS: modified Rankin Scale; NIHSS: National Institutes of Health Stroke Scale; TOAST: Trial of Org 10172 in Acute Stroke Treatment; UA: urinalysis; UTI: urinary tract infection

	No UTI	All UTI	Community-acquired UTI	Hospital-acquired UTI		
Variable	N (%)	N (%)	OR (95% CI)*	N (%)	N (%)	OR (95% CI)*
Total	349 (88.4)	46 (11.6)		30 (65.2)	16 (34.8)	
Age (years)						
Q1: ≤ 58	98 (95.1)	5 (4.9)	Reference	2 (40.0)	3 (60.0)	Reference
Q2: 59-72	92 (91.1)	9 (8.9)	1.92 (0.62, 5.93)	3 (33.3)	6 (66.7)	1.33 (0.14, 12.81)
Q3: 73-81	80 (84.2)	15 (15.8)	3.67 (1.28, 10.54)	9 (60.0)	6 (40.0)	0.44 (0.06, 3.51)
Q4: > 81	79 (82.3)	17 (17.7)	4.22 (1.49, 11.93)	16 (94.1)	1 (5.9)	0.04 (0.0, 0.62)
Gender						
Female	150 (82.4)	32 (17.6)	3.03 (1.56, 5.88)	19 (59.4)	13 (40.6)	2.51 (0.58, 10.79)
Male	199 (93.4)	14 (6.6)	Reference	11 (78.6)	3 (21.4)	Reference
Admission Source						
Home	266 (92.0)	23 (8.0)	Reference	18 (78.3)	5 (21.7)	Reference
Outside hospital	16 (64)	14 (17.3)	2.42 (1.18, 4.95)	9 (100.0)	11 (78.6)	12.06 (2.14, 95.32)
Nursing home	67 (82.7)	9 (36.0)	6.51 (2.59, 16.34)	3 (21.4)	0 (0.0)	0.33 (0.0, 2.01)
Foley						
Yes	98 (80.3)	24 (19.7)	2.65 (1.41, 4.98)	13 (54.2)	11 (45.8)	2.71 (0.75, 9.80)
No	227 (91.5)	21 (8.5)	Reference	16 (76.2)	5 (23.8)	Reference
UA positive results						
Positive	212 (95.1)	34 (65.4)	36.40 (15.83, 83.72)	23 (67.7)	11 (32.4)	0.84 (0.20, 3.47)
Negative	18 (34.6)	11 (4.9)	Reference	7 (63.6)	4 (36.4)	Reference
TOAST criteria						
Large vessel	102 (90.3)	11 (9.7)	0.53 (0.23, 1.21)	5 (45.5)	6 (54.6)	3.14 (0.49, 23.45)
Small vessel	92 (90.2)	10 (9.8)	0.53 (0.22, 1.25)	7 (70.0)	3 (30.0)	1.17 (0.13, 9.50)
CardioEmbolic	73 (83.0)	15 (17.1)	Reference	11 (73.3)	4 (26.7)	Reference
Other determined	23 (85.2)	4 (14.8)	0.85 (0.26, 2.81)	1 (25.0)	3 (75.0)	7.25 (0.44, 471.39)
Undetermined	59 (90.8)	6 (9.2)	0.50 (0.18, 1.36)	6 (100.0)	0 (0.0)	0.40 (0.0, 2.72)
Admission severe stroke (NIHSS ≥ 10)						
Yes	69 (87.3)	10 (12.7)	1.12 (0.53, 2.38)	6 (60.0)	4 (40.0)	1.39 (0.33, 5.97)
No	263 (88.6)	34 (11.5)	Reference	23 (67.7)	11 (32.4)	Reference
Admission poor condition (mRS ≥ 3)						
Yes	76 (90.5)	4 (17.4)	1.65 (0.53, 5.09)	3 (75.0)	1 (12.5)	0.21 (0.02, 1.94)
No	257 (98.0)	40 (11.3)	Reference	26 (65.0)	14 (40.0)	Reference

Patients admitted from another hospital were more likely to be diagnosed with a UTI compared to those arriving from home (OR 2.42 95% CI 1.18, 4.95). Similarly, those with a Foley catheter were also more likely to be diagnosed with a UTI (OR 2.65 95% CI 1.41, 4.98). As expected, since it is one of the diagnostic tests for UTI, positive UA results were strongly associated with the diagnosis of UTI (as defined as a positive urine culture). Stroke severity (admission NIHSS) and preadmission condition (preadmission mRS) were not associated with UTI. When comparing CA-UTI versus HA-UTI, those patients admitted from an outside hospital were considerably more likely to develop an HA-UTI than a CA-UTI (OR 12.06 95% CI 2.14, 95.32). For patients admitted from a nursing home the finding, although not statistically significant, was reversed. Patients in the highest age quartile (> 81) were significantly less likely to suffer from a HA-UTI as opposed to a CA-UTI (OR 0.04 95% CI 0.0, 0.62). Stroke severity and preadmission condition (as defined by preadmission mRS) were not significantly associated with either CA-UTI or HA-UTI. 

A bivariate analysis of the factors associated with the clinical condition on discharge, the length of stay, and hospital charges can be seen in Table 3. 

**Table 3:** Factors Associated with Poor Clinical Outcome, Length of Stay, and Hospital Charges † Odds ratios and p-values from logistic regression, odds ratios adjusted for all other variables in the table ‡ Means, 95% confidence limits, and pairwise comparisons from Analysis of Variance (ANOVA), means adjusted for all other variables in the table mRS: modified Rankin Scale; NIHSS: National Institutes of Health Stroke Scale; TOAST: Trial of Org 10172 in Acute Stroke Treatment; UA - urinalysis; UTI: urinary tract infection

Variable	Discharge Poor Condition †	Length of Stay (days) ‡	Hospital Charges ($) ‡			
	(N = 395)	(N = 373)	(N = 373)			
	N (%)	OR (95% CI)	Mean (95% CI)	Differences	Mean (95% CI)	Differences
Any UTI						
Yes	19 (41.3)	1.57 (0.84, 2.95)	5.51 (4.51, 6.73)	Yes > No	13100 (10884, 15768)	Yes > No
No	108 (31.0)	Reference	4.18 (3.88, 4.51)		10523 (9817, 11281)	
UTI Type						
None	108 (31.0)	Reference	4.18 (3.88, 4.50)	Hosp > Comm	10523 (9820, 11277)	Hosp > None
Hospital-acquired	6 (37.5)	1.33 (0.48, 3.78)	8.08 (5.78, 11.31)	Hosp > None	17354 (12694, 23725)	
Community-acquired	13 (43.3)	1.71 (0.80, 3.64)	4.49 (3.51, 5.74)		11276 (8974, 14169)	
Age (years)						
Q1: ≤ 58	29 (28.2)	Reference	4.50 (3.91, 5.18)		12414 (10912, 14124)	
Q2: 59-72	32 (31.7)	1.18 (0.65, 2.16)	4.22 (3.67, 4.85)		10738 (9451, 12201)	
Q3: 73-81	23 (24.2)	0.82 (0.43, 1.54)	4.23 (3.66, 4.89)		10143 (8883, 11581)	
Q4: > 81	43 (44.8)	2.07 (1.15, 3.73)	4.37 (3.78, 5.04)		10041 (8801, 11456)	
Gender						
Female	57 (31.3)	0.93 (0.61, 1.42)	4.68 (4.22, 5.18)	Female > Male	11173 (10161, 12286)	
Male	70 (32.9)	Reference	4.03 (3.66, 4.44)		10495 (9589, 11486)	
Admission Source				OSH > Home		OSH > Home
Home	74 (25.6)	Reference	3.92 (3.62, 4.25)		9820 (9113, 10582)	
Outside hospital (OSH)	34 (42.0)	2.10 (1.26, 3.52)	5.69 (4.91, 6.61)		13095 (10136, 16919)	
Nursing home	19 (76.0)	9.20 (3.54, 23.91)	5.29 (4.01, 6.99)		14156 (12339, 16241)	
Foley						
Yes	73 (59.8)	6.05 (3.75, 9.76)	5.97 (5.29, 6.74)	Yes > No	15927 (14309, 17727)	Yes > No
No	49 (19.8)	Reference	3.70 (3.40, 4.03)		9050 (8390, 9762)	
UA positive results						
Positive	23 (44.2)	1.73 (0.94, 3.21)	5.68 (4.66, 6.93)	Pos > Neg	13738 (11428, 16515)	Pos > Neg
Negative	70 (31.4)	Reference	4.40 (4.00, 4.85)		11198 (10246, 12240)	
TOAST criteria						Cardio > Small
Large vessel	41 (36.3)	0.60 (0.34, 1.05)	5.13 (4.52, 5.82)	Cardio > Small	12946 (11539, 14525)	Large > Small
Small vessel	14 (13.7)	0.17 (0.08, 0.34)	3.34 (2.93, 3.82)	Large > Small	8151 (7223, 9199)	Large > Undet.
CardioEmbolic	43 (48.9)	Reference	4.75 (4.11, 5.48)	Large > Undet.	11459 (10062, 13050)	Other > Cardio
Other Determined	12 (44.4)	0.84 (0.35, 1.99)	6.59 (5.07, 8.56)	Other > Small	18399 (14519, 23317)	Other > Small
Undetermined	17 (26.2)	0.37 (0.19, 0.74)	3.60 (3.06, 4.25)	Other > Undet.	9209 (7933, 10691)	Other > Undet.
Admission severe stroke (NIHSS ≥ 10)						
Yes	60 (76.0)	13.89 (7.68, 25.15)	6.62 (5.72, 7.66)	Yes > No	17862 (15726, 20288)	Yes > No
No	55 (18.5)	Reference	3.78 (3.50, 4.08)		9251 (8646, 9898)	
Admission poor condition (mRS ≥ 3)						
Yes	58 (69.1)	9.61 (5.56, 16.62)	5.42 (4.67, 6.30)	Yes > No	13508 (11770, 15503)	Yes > No
No	55 (18.8)	Reference	3.98 (3.66, 4.31)		10011 (9289, 10789)	

As expected, older age, a more severe stroke and a poor condition on admission were all associated with higher odds of a poor outcome. However, while the presence of a Foley catheter was also associated with a poor outcome, the presence of a UTI itself was not. The presence of a UTI of any type was associated with an increased LOS and increased hospital charges. Patients with a HA-UTI had a longer length of stay than both CA-UTI patients and those patients without a UTI of any type. Again, illness severity in terms of preadmission mRS and admission NIHSS were associated with longer LOS and greater hospital charges. 

Finally, Table 4 shows the results of a multivariate analysis of factors associated with clinical outcome, LOS, and hospital charges while adjusting for all other co-factors. 

**Table 4:** Factors Associated with Poor Clinical Outcome, Length of Stay, and Hospital Charges Adjusted for All Other Factors * Any UTI is adjusted for all variables included the table but the estimates for the other variables are from the model including UTI type † Odds ratios and p-values from logistic regression, odds ratios adjusted for all other variables in the table ‡ Means, 95% confidence limits, and pairwise comparisons from Analysis of Variance (ANOVA), means adjusted for all other variables in the table mRS: modified Rankin Scale; NIHSS: National Institutes of Health Stroke Scale; TOAST: Trial of Org 10172 in Acute Stroke Treatment; UA - urinalysis; UTI: urinary tract infection

Variable	Discharge Poor Condition †	Length of Stay (days) ‡	Hospital Charges ($) ‡			
	(N = 247)	(N = 232)	(N = 232)			
	N (%)	OR (95% CI)	Mean (95% CI)	Differences	Mean (95% CI)	Differences
Any UTI*						
Yes	15 (37.5)	0.51 (0.14, 1.89)	5.58 (4.36, 7.14)		14526 (11843, 17817)	
No	65 (31.4)	Reference	5.50 (4.45, 6.80)		14910 (12514, 17763)	
UTI Type						
None	65 (31.4)	Reference	5.60 (4.53, 6.92)		14989 (12567, 17878)	
Hospital-acquired	4 (30.8)	0.48 (0.08, 2.91)	7.31 (4.83, 11.07)		15805 (11200, 22304)	
Community-acquired	11 (40.7)	0.53 (0.12, 2.35)	4.98 (3.75, 6.61)		14021 (11082, 17740)	
Age (years)						
Q1: ≤ 58	20 (29.4)	Reference	6.27 (4.87, 8.07)		16911 (13715, 20852)	
Q2: 59-72	14 (25.0)	1.08 (0.36, 3.24)	5.83 (4.54, 7.50)		15526 (12604, 19127)	
Q3: 73-81	15 (23.8)	1.01 (0.36, 2.84)	5.54 (4.30, 7.15)		13694 (11085, 16919)	
Q4: > 81	31 (51.7)	2.61 (0.85, 8.00)	5.92 (4.67, 7.51)		13783 (11310, 16797)	
Gender						
Female	41 (33.3)	0.67 (0.30, 1.50)	6.28 (5.14, 7.67)		14814 (12551, 17484)	
Male	39 (31.5)	Reference	5.52 (4.39, 6.93)		15028 (12434, 18163)	
Admission Source						
Home	47 (27.2)	Reference	5.66 (4.57, 7.02)		14753 (12347, 17627)	
Outside hospital	21 (36.8)	2.21 (0.34, 14.54)	6.30 (5.05, 7.85)		14616 (12169, 17555)	
Nursing home	12 (70.6)	0.73 (0.28, 1.93)	5.72 (3.90, 8.37)		15405 (11224, 21143)	
Foley						
Yes	52 (56.5)	2.34 (1.02, 5.36)	6.73 (5.49, 8.24)	Yes > No	17962 (15175, 21260)	Yes > No
No	28 (18.1)	Reference	5.15 (4.07, 6.52)	P = 0.014	12394 (10186, 15081)	P < 0.001
UA positive results						
Positive	19 (41.3)	3.13 (0.96, 10.20)	6.42 (5.00, 8.24)		16258 (13211, 20007)	
Negative	61 (30.4)	Reference	5.39 (4.22, 6.89)		13693 (11176, 16778)	
TOAST criteria						
Large vessel	26 (38.2)	0.56 (0.21, 1.47)	6.66 (5.28, 8.40)		16529 (13636, 20034)	Large > Small
Small vessel	7 (11.9)	0.16 (0.05, 0.54)	4.81 (3.74, 6.18)		11712 (9508.0, 14426)	P = 0.01
CardioEmbolic	31 (50.8)	Reference	5.67 (4.48, 7.18)		13923 (11445, 16938)	
Other Determined	8 (44.4)	1.35 (0.32, 5.75)	7.11 (4.93, 10.24)		20195 (14907, 27358)	Other > Small
Undetermined	8 (19.5)	0.35 (0.11, 1.14)	5.47 (4.12, 7.27)		13586 (10737, 17191)	P = 0.007
Admission severe stroke (NIHSS ≥ 10)						
Yes	41 (75.9)	6.23 (2.33, 16.62)	6.84 (5.32, 8.79)	Yes > No	18305 (14855, 22557)	Yes > No
No	39 (20.2)	Reference	5.07 (4.13, 6.22)	P = 0.017	12162 (10261, 14415)	P < 0.001
Admission poor condition (mRS ≥ 3)						
Yes	46 (75.4)	8.45 (3.56, 20.02)	6.00 (4.70, 7.67)		14710 (12002, 18029)	
No	34 (18.3)	Reference	5.77 (4.72, 7.05)		15134 (12816, 17870)	

Two separate statistical models were used. In the first, any UTI was modeled with the other covariates (first row), while in the second, UTI type (none, CA-UTI, or HA-UTI) was modeled (all other rows include results from this model). After adjusting for the various covariates, we did not find a statistically significant relationship between either any UTI or a specific UTI subtype and discharge mRS, LOS, or hospital charges. However, the presence of a Foley catheter was associated with poor clinical outcome (adjusted odds ratio (aOR) 2.34 95% CI 1.02, 5.36), increased LOS (6.73 days versus 5.15 days, p = 0.04), and increased hospital charges ($17,962 versus $12,394, p < 0.001). Admission stroke severity remained associated with higher odds of discharge in poor condition (aOR 6.23 95% CI 2.33, 16.62), a longer number of days LOS (6.84 versus 5.07, p = 0.017) and higher hospital charges ($18,305 vs $12,162, p < 0.001).  

## Discussion

The role of infection in ischemic stroke is likely to be complex and multifactorial. Infection may play a causal role in the immunological triggering of stroke, and a stroke itself may have untoward effects on the immune system. Syrjänen, et al. found increased serum bacterial antibody levels in young patients with acute stroke compared to controls [12]. Subsequent papers also reported that infection is a risk factor for stroke with up to 25-35% of stroke patients having infections preceding their stroke [13-14]. The concepts of central nervous system (CNS) injury-induced immunodepression or stroke-induced immunodepression have even been used to describe the findings of secondary immunodeficiency after stroke [15-16]. While therapeutic immunomodulation as a treatment for stroke has not resulted in clear outcome improvement in humans, at least one study focusing on prophylactic antibiotic therapy for acute stroke yielded promising results [17]. UTI, in particular, is one of the most common infections seen in stroke patients. In addition, unlike other infections such as pneumonia or invasive line infections, UTI are often present on admission, which complicates efforts at eradication. Given its frequency, it is therefore not surprising that detection of CA-UTI and a reduction in HA-UTI have been the focus of numerous QI initiatives. However, it is not clear that the early detection and treatment of a CA-UTI or the later finding of a HA-UTI by themselves portend a poor outcome. Rather, since many UTIs are minimally symptomatic and, in general, are easily and inexpensively treated, the overall impact on a complex disease, such as stroke, may be minimal.

In an effort to better understand the role the diagnosis of UTI plays in the course of an ischemic stroke patient, we examined the incidence and implications of a UTI diagnosis on all of the ischemic stroke patients admitted to a single institution over a two-year period. We defined a UTI as a positive urine culture since our practice is to treat with antibiotics only those patients that have a positive culture. Data collected included the frequency of urinary catheter placement, UA results, and clinical outcome at discharge, as well as information on length of stay (LOS) and hospital charges. The rate of UTI in our population was approximately 12% with majority (65%) being found on admission (CA-UTI). Previous studies in stroke patients have quoted a wide range of rates for UTI, from as low as 3% up to as high as 44% [18]. Much of this variability can be explained by the length of follow-up, with a higher rate of UTI found in populations with longer follow-up periods. In addition, our HA-UTI rate was 4.1%, considerably lower than that reported in two older studies from 2000 and 2009 (16% and 24%) [5, 19]. It is possible that this reduction in the HA-UTI rate may be due to QI initiatives launched over the past few years, but unfortunately, robust data from earlier years is not available. 

In our analysis, older patients were more likely to be found to have a UTI. In the oldest age group (> 81 years of age), patients were also significantly more likely to have come from a nursing home. This association can be explained by the fact that patients in nursing homes tend to have a higher incidence of infections, such as UTI, as well as being older with more comorbidities compared to patients residing at home [20]. It should be noted that although older people may be more likely to get a HA-UTI, they are also more likely to have a CA-UTI; in this study design, patients cannot crossover from a CA-UTI to a HA-UTI. For the TOAST criteria subcategories, large vessel, cardioembolic, and other determined had a higher incidence of poor mRS. These findings correlate with previous studies that have shown a worse outcome with these categories compared to others, such as a small vessel [21-23]. Urinary catheters were also a risk factor for UTI, as has been studied extensively [24]. As expected, overall UA results were associated with UTI as defined as a positive urine culture. 

While overall rates of UTIs, CA-UTIs, and HA-UTIs were relatively low, we were most interested in whether these infections had any impact on the outcome. To assess clinical outcome, we chose to use the patient’s clinical status (mRS) at discharge, since we felt that this earlier time point would be more likely to be impacted by an event during the patient’s hospital course and that patients themselves would be most interested in their functional status. We included LOS and hospital charges as these parameters are frequently used as benchmarks in QI initiatives. Confirming the representativeness of our stroke population, our multivariable analysis found that stroke severity and preadmission clinical condition were strongly correlated with the clinical condition at discharge. As expected, patients with more severe strokes also had longer hospital stays and generated more hospital charges. However, importantly, this same analysis did not find a statistically significant association between UTI in general or a specific type of UTI (CA-UTI vs HA-UTI) and any of the three outcome measures studied. This finding does not mean that it should be concluded that UTI in stroke patients should not be treated, rather this finding suggests that QI efforts to eradicate UTI should be viewed realistically in terms of their overall impact on the stroke patient. It would appear that even if expensive and time-consuming QI efforts are successful in eliminating HA-UTI, such efforts would be unlikely to improve the clinical outcome of stroke patients and may not produce a substantial cost saving for the healthcare facility either. In the end, UTI may be a small piece of the overall picture in the recovery of the complicated stroke patient.

Obviously, this may be a controversial assertion and previous reports have shown conflicting results regarding the impact of UTI on stroke patients. While some studies have found a correlation between UTIs and poor outcome [4, 25], others have not [19]. A study by Tirschwell, et al. [26] found a 40% increased the length of stay in stroke patients with UTI. However, although age and gender were controlled for, stroke severity was not. In addition, CA-UTI and HA-UTI were not separately assessed. Aslanyan, et al. [4] showed an association after multivariate analysis of UTI and poor outcome by mRS at three months, but again, this study did not distinguish between CA-UTI and HA-UTI. On the other hand, an initial association of UTI with death and disability found by Stott, et al. [19] on univariate analysis failed to remain significant after controlling for stroke severity and pre-stroke morbidity. To our knowledge, this is the first study to examine the impact of UTI on mRS, the length of stay, and hospital charges in stroke patients while simultaneously differentiating between CA-UTI versus HA-UTI. 

The limitations of this study, as with other studies that have looked at this subject, include the inherent problems and biases of a retrospective design. Since follow-up is limited to hospital discharge, it is possible that there may have been a delayed effect of UTI on the outcome, although this would seem to be unlikely as most stroke patients who survive their initial hospitalization tend to improve. It is also possible that the sample size was too small to detect a statistically significant difference. However, the finding that during two years at a reasonably busy Joint Commission-certified Primary Stroke Center, the occurrence of a UTI of any type had no independent impact on clinical outcome remains clinically relevant. In terms of the outcome measures, it may be argued that the mRS is not sensitive enough; however, for the patient, the presence or absence of disability is vitally important. The length of stay and hospital charges may have varying relevance to the patient, but they are commonly used as metrics to assess the impact of QI programs.

## Conclusions

Urinary tract infections remain a common occurrence in stroke patients. In our study, the majority of UTIs were present on admission, which makes admission screening an important component of any hospital QI program where UTIs are concerned. However, after adjusting for stroke severity and preadmission condition, we did not find a significant association between UTI of any type and early clinical condition, the length of stay, or hospital charges. These results suggest that QI initiatives focusing on the prevention and treatment of hospital-acquired UTIs may have minimal impact on traditional outcome benchmarks.
